# Environmental complexity impacts anxiety in broiler chickens depending on genetic strain and body weight

**DOI:** 10.1038/s41598-024-67965-z

**Published:** 2024-07-30

**Authors:** Alexandra Ulans, George C. Brooks, Leonie Jacobs

**Affiliations:** 1 School of Animal Sciences, Virginia Tech, Blacksburg, VA 24061 USA; 2 Department of Fish and Wildlife Conservation, Virginia Tech, Blacksburg, VA 24061 USA

**Keywords:** Agricultural genetics, Emotion, Attention

## Abstract

The objective was to assess the impact of environmental complexity on affective state (anxiety) in fast- and slow-growing broilers (*Gallus gallus domesticus*) as they gain weight. Six hundred fast-growing broilers (Ross 708; “fast-growers”) and 600 slow-growing broilers (Hubbard Redbro Mini; “slow-growers”) were raised in 24 pens with simple (standard; SE) or complex (permanent and temporary enrichments; CE) environments. Six birds/pen underwent the attention bias test on day 23 (fast-growers only), 28–29, 35–36, 42–43, and 56–57 (slow-growers only), with individuals only tested once (n = 576). Proportion of birds feeding, time spent vigilant and latencies to eat and step were recorded. Greater vigilance and longer latencies indicate more anxiety. Slow-growers fed more (p = 0.001), were less vigilant (p = 0.003), and stepped sooner than fast-growers (p = 0.007). For both strains, likelihood of feeding was unrelated to weight in SE, but decreased with increasing weight in CE (p = 0.048). Birds in CE stepped sooner than birds in SE (p = 0.030). Vigilance increased with body weight (p = 0.024). These results indicate that affective state (anxiety) can change as birds gain weight, depending on environmental complexity and genetic strain. Overall, slow-growers showed reduced anxiety compared to fast-growers, across housing treatments or weights.

## Introduction

Broiler chickens have been genetically selected for fast growth, improved feed conversion, and large breast muscles^1^. However, selection for these production-related traits can result in poor animal welfare outcomes. Commonly used fast-growing strains have a higher prevalence of sudden death syndrome, ascites, leg disorders, bone deformations, and mortality rates compared to slow-growing strains^2–5^. Furthermore, fast-growing broilers show adapted behavioral repertoires with more frequent sitting, feeding, and drinking and less frequent foraging, preening, dust bathing, and perching^6,7^. As growth rates and body weights increase with age, animal welfare status can also change. Conventional fast-growing broilers show deteriorated walking ability as they age, with worst gait scores at processing age compared to earlier ages (day 47^8^; day 54^9^), and worsened footpad dermatitis lesions with age^10^. Furthermore, they spend less time walking, running, foraging, and preening by week 5 compared to earlier weeks^11,12^, and less time being active at 4 and 7 weeks of age compared to 2 weeks of age^13^, likely due to the weight gain hampering their ability to perform these behaviors.

Slow-growing broilers can show improved welfare outcomes related to health and behavior compared to fast-growing strains^6,7^. The threshold for strains to be considered slow-growing differs among stakeholders, with 43–47 g/day^14^ or < 50 g/day commonly chosen^7,15^. Slow-growing broilers had lower mortality and cull rates, better leg and foot health, and higher incidences of behaviors indicative of good welfare compared to fast-growing broilers^7,16^. However, fast-growing birds had a shorter avoidance distance than slow-growing broilers, indicating that fast-growing broilers show reduced fear of humans, although neophobia did not differ^16^. While this outcome implies that fast-growing broilers may experience less fear of humans than slow-growing broilers, the authors acknowledge that these results may be due to the fast-growing broilers being less physically able to express fear or less motivated to move. Thus, the majority, if not all, results indicate that slow-growing broilers have improved welfare compared to fast-growing birds.

As the current commercial infrastructure is set up for fast-growing broilers, increasing environmental complexity could be a means to improve welfare for fast-growing broilers in the short-term. Access to perches and platforms was associated with improved leg strength^17^, plumage cleanliness, reduced hock burn and footpad dermatitis severity^18^, and decreased disturbances^19^ compared to conventionally-housed broilers. Panels, barriers, and bales of substrate improved leg strength and bird distribution throughout the house, stimulated perching, reduced disturbances^19^, and decreased severity of footpad dermatitis and lameness compared to broilers housed without those enrichments^20–23^.

Besides behavior and health outcomes, affective state may be impacted by housing conditions. Affective state is the accumulation of emotions and moods experienced by the animal, and can range from positive to negative depending on the valence of the accumulated experiences^24^. Resources like hanging bundles of strings, treat dispensing toys, plastic balls, hanging heads of lettuce, plastic bottles, and mirrors reduced fear^25–28^, anxiety^26^, and increased optimism^29^ in fast-growing broiler chickens. These benefits may be similar for fast- and slow-growing strains, but this is unknown.

We should determine if growth rate and environmental complexity impact affective states. Some laying hen strains experienced greater fear and stress when compared to other strains, when other factors were controlled for^30–35^. This may be due to genetic selection for low reactivity in production systems that require greater interaction with humans compared to those with less^35^. The impact of genetic strain on affective states in broilers is currently unknown.

An attention bias test is used to determine anxiety in chickens, which is a negatively-valenced affective state^29,36–38^. In this test, anxiety is determined based on the time it takes for the chicken to change the focus of their attention away from the negative stimulus and towards the positive stimulus. A prolonged focus on the negative stimulus is indicative of greater levels of anxiety, thus a more negative affective state^37^.

Slow-growing strains are becoming more common in commercial production^15,39^. Consumers show concern about the treatment of production animals and their welfare status, and report a willingness to pay for welfare improvements^40,41^. For instance, when consumers are provided news articles with professionals stating the improved health of slow-growing broilers, they report a willingness to pay a premium for products from slow-growing broilers compared to products from fast-growing broilers^42^. Voluntary animal welfare certification programs in the United States will require slow-growing strains for their suppliers^43,44^. Slower-growing broiler strains comprise almost 40% of Dutch broiler production, 24% of French broiler production, and 11% of production in the United Kingdom^45^. Due to this increase in slow-growing genetics, more insight in the animal welfare experience of different genetic strains is valuable to ensure optimal animal welfare outcomes. While differences in health and behavior for fast-growing and slow-growing broilers have been compared previously, no studies compared emotion and affective states between strains besides fear. No studies have addressed how affect changes as either fast- or slow-growing broilers age and gain weight. Furthermore, the impact of environmental complexity can play a role in these outcomes. Therefore, our objective was to determine how fast-growing and slow-growing broilers’ anxiety change as they gain weight and age, when housed in complex (CE) or simple (SE) housing conditions. We hypothesized that fast-growing broilers and broilers raised in a simple environment will be more anxious than slow-growing birds or birds in a complex environment. We also hypothesized that anxiety will increase as birds age and gain weight in fast-growing broilers, but not in slow-growing broilers.

## Methods

### Ethics declarations

This experiment was carried out at Virginia Tech’s Turkey Research Center from March through May 2022. This study was approved by the Virginia Tech Institutional Animal Care and Use Committee (protocol number 21-221), all methods were performed in accordance with relevant guidelines and regulations, and are reported according to the ARRIVE guidelines.

### Animals and housing

This experiment involved a 2 × 2 factorial approach in a randomized block design with broiler chicken strain and environmental complexity as factors at pen-level. Six-hundred male Ross 708 (fast-growing strain) and 600 male Hubbard Redbro Mini (slow-growing strain) day-old chicks were obtained from a hatchery (South Fork, PA, USA) where they were vaccinated for Marek’s disease and then transported to the research facility. Chicks were separated by strain and chicks were arbitrarily distributed across 24 pens upon arrival, with 50 chicks per pen, resulting in 6 replicates per treatment. Pens (3.5 × 2.5 m) contained new pine shavings at approximately ~ 6 cm in depth, with three hanging galvanized tube feeders and two water lines with three nipple drinkers each. The chickens were able to see and hear conspecifics in other pens, which could potentially allow for emotional contagion^46^. Fast-growing broiler pen stocking densities were calculated on days 16, 30, and 44 and were (mean ± SD) 3.09 ± 0.04, 9.71 ± 0.12, 17.71 ± 0.14 kg/m^2^ respectively. Slow-growing broiler pen stocking densities were calculated on days 22, 44, and 53 of age and were 3.88 ± 0.06, 12.14 ± 0.28, and 16.12 ± 0.66 kg/m^2^, respectively. The birds had ad libitum access to feed and water. The corn-soybean meal diets were the same for both strains and were prepared according to the nutritional specifications for conventional broiler chickens split into three feeding phases: starter (3000 kcal ME and 23% CP), grower (3100 kcal ME and 21.5% CP), and finisher (3150 kcal ME and 20% CP)^47^. Fast-growing broilers received starter from day 0–16, grower from day 16–30, and finisher from day 30–44. Slow-growing broilers received starter from day 0–22, grower from day 22–44, and finisher from day 44–58. The house was heated to 35 °C on day 0 and was gradually reduced to 21 °C by day 29, and remained at 21 °C until the end of the trial. Broilers were kept in an artificial lighting schedule of 24L:0D in the first 4 days due to heat lamps, then 20L:4D from day 5 to 7, and 18L:6D from day 8 until the end of the experiment, with a light intensity of approximately 15 lx during light hours.

### Genetic strain

Ross 708 broilers are a white-feathered fast-growing broiler chicken strain, reaching a live body weight of approximately 3 kg by day 42 with an average daily gain of 68.1 g^48^. The fast-growing birds in this experiment reached a mean live body weight of 3.10 kg by day 44 with an average daily gain of 69.6 g. Hubbard Redbro Mini broilers are a brown-feathered slow-growing broiler chicken strain, reaching a mean live body weight of 3.06 kg by day 58, with an average daily gain of 52.1 g. Each strain was randomly assigned to 12 of 24 pens.

### Environmental complexity

**Figure 1:**
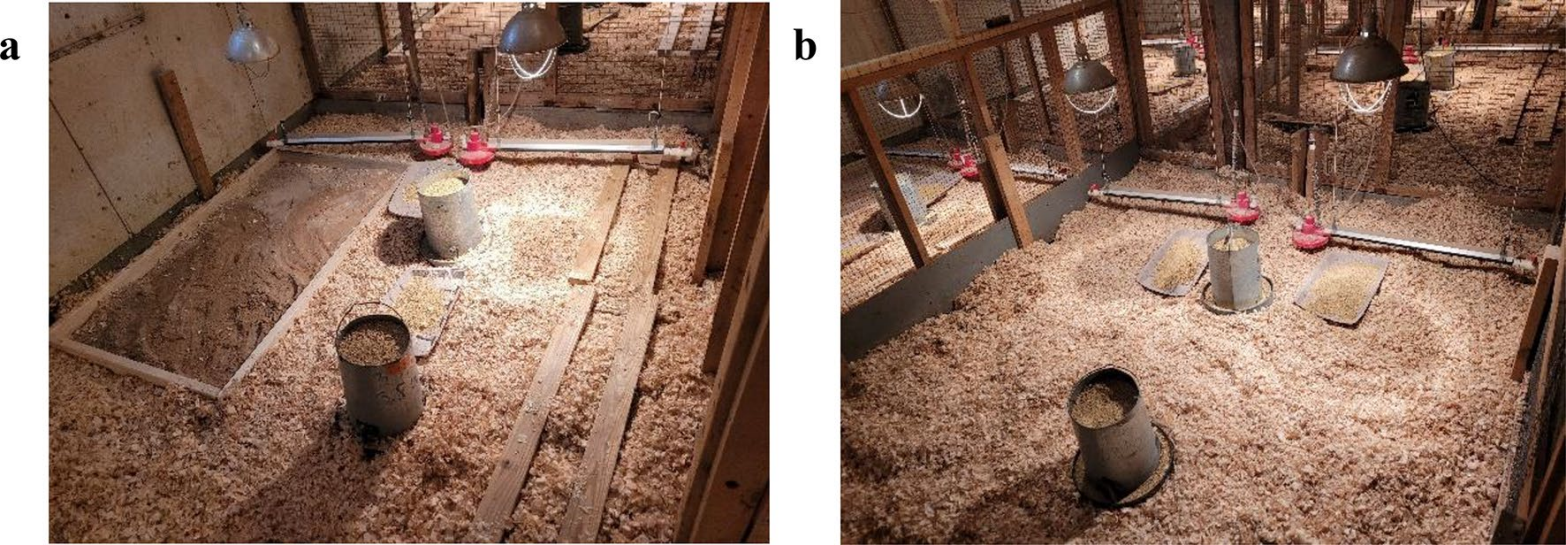
Photograph of (**a**) complex environment pen with permanent enrichments including a wooden dust bath with sand and two wooden platform perches, and (**b**) a simple environment pen without enrichments.

Broilers were housed in pens with either complex or simple environments (Fig. 1). The complex environment pens contained permanent and temporary enrichments. Permanent enrichments consisted of one wooden dust bath (180 cm L × 91 cm W × 10 cm H) filled with playground sand (Quikrete, Atlanta, GA, USA) and two wooden perches (488 cm L × 46 cm W × 10 cm H each) providing 19.5 cm flat surface perch space per broiler (Fig. 1a). Six temporary enrichments were rotated every 3 days with one set of two enrichments in the pen at one time. These enrichments include two treat balls (Lixit Corp., Napa, CA, USA) filled with a handful of oats (Red Mill, Milwaukie, OR, USA) and three hanging bundles of string, half a cabbage hung at bird height and alfalfa hay provided in two metal cage balls (Darice, Strongsville, OH, USA), or two hanging mirrors (19 × 28 cm) and a handful of chicken scratch (MannaPro, Chesterfield, MO, USA) thrown into the litter. Nutritional enrichments were refilled or replaced as needed during the morning or evening health check. Simple environment pens did not contain any enrichments (Fig. 1b). Each complexity treatment was randomly assigned to 12 of 24 pens.

### Measurements

All birds were wing-banded on day 3 of age for identification throughout the experiment. Focal birds were marked with livestock paint (All-Weather Paintstik, LA-CO Industries, Inc., Elk Grove Village, IL, USA) for identification during tests. Measurements were performed at three age-matched and three weight-matched timepoints, where broilers were either 4, 5, and 6 weeks of age or weighed 1, 2, and 3 kg in live body weight. Birds were never tested twice to avoid habituation to the test. Six focal birds were individually weighed prior to testing to assure target weights were met, allowing 10% variation (Table 1). Fast-growing broilers at the 1-kg and 2-kg timepoints exceeded this goal, resulting in 16.3% and 11.2% variation respectively. Some age and weight timepoints overlapped, resulting in a total of four sampling timepoints per genetic strain (Table 2).

**Table 1:** Live body weight (mean ± standard deviation) of tested birds at each weight timepoint per genetic strain.

Weight timepoint	Body weight (kg)	
	Fast-growing strain	Slow-growing strain
1 kg	0.98 ± 0.16	1.06 ± 0.09
2 kg	1.96 ± 0.22	1.97 ± 0.15
3 kg	2.89 ± 0.26	2.94 ± 0.22

**Table 2:** Broilers’ age at time of testing at the weight-balanced timepoints or age-balanced timepoints per genetic strain.

Timepoint	Age (days)	
	Fast-growing strain	Slow-growing strain
Weight point	22, 34, 41	27, 41, 56
Age point	27, 34, 41	27, 34, 41

These birds were part of a larger study. All sampled birds reported here, additionally underwent a tonic immobility test, and were handled for the collection of feathers (cut with scissors at the calamus, assumed to be painless). Those results will be reported elsewhere. Other birds, not the same as reported on here, underwent a suite of behavioral assessments, as reported in^49^.

### Bird selection

Six focal birds per pen (n = 144; 72 per genetic strain and complexity treatment) were arbitrarily selected at each sampling timepoint. However, broilers were gait scored^50^ prior to selection so that lame birds were not tested.

### Attention Bias Test (ABT)

The Attention Bias Test (ABT) was used to measure anxiety, an affective state^37^. The ABT was performed at a group-level as described by^26,36^, by four observers. The ABT was performed with three familiar birds per test, in an arena placed in a separate room in the facility. The positive stimulus was presented in the center of the arena and consisted of familiar feed and dried mealworms. All three birds were placed in quick succession, and after the third bird was placed, a negative stimulus was presented for 8 s, which consisted of a conspecific alarm call signaling a ground predator (recorded by^37^) using a portable speaker at approximately 95 Db (FUGOO, Van Nuys, Irvine, CA, USA). This alarm call elicits a vigilance response in chickens^26,37,51^. Latencies to begin and resume feeding were recorded, as was the response rate (proportion of birds feeding). A longer latency to begin and resume feeding is indicative of increased anxiety, thus a more negative affective state^26,37^. After the initial call was played, the alarm call was replayed if two of four scenarios occurred:

- If all three birds began to feed, latency was recorded, and the last bird was allowed to feed for 5 s. Then, the alarm call was replayed and latency to resume feeding was recorded for all birds. The maximum duration to resume feeding was 120 s and birds that did not resume feeding did not receive a score.
- If two birds began to feed, the alarm call was replayed at 300 s and latency to resume feeding was recorded for the two birds that fed. The third bird received a latency to begin feeding of 300 s. The maximum duration to resume feeding was 120 s and birds that did not resume feeding did not receive a score.
- If one bird began to feed, the alarm call was not replayed, and the test ended after 300 s.
- If no birds began feeding, we recorded a latency to begin feeding of 300 s for all three birds and the test ended after 300 s.

Latency to begin feeding (sec), latency to resume feeding (sec), and the response rate (proportion of birds feeding) after the second alarm call were recorded. Tests were video recorded (EOS Rebel T7 DSLR Camera, Canon, Melville, NY, USA). Duration of vigilance behaviors from one randomly selected bird out of the three birds in a test was scored using continuous focal animal sampling from the start of the test until the alarm call was replayed, or until the test ended if the alarm call was not replayed. A bird was considered vigilant if at least one of the behaviors (Table 3) was observed. If a selected bird was out of view for > 20% of the test (n = 43 out of 177 birds), another bird (out of the remaining two) was randomly selected and observed. Only one of three birds were selected to assess vigilance behavior due to time constraints. Behaviors (Table 3) were coded by a single trained observer using BORIS software^52^.

**Table 3:** The ethogram used to score vigilance behaviors within the attention bias test.

Behavior	Description
Freeze	The bird holds the same position without movement. Freezing is considered over once the bird moves a part of its body (head, neck, legs, wings, etc.)^103,104^
Erect posture	The bird stands upright, holding its head high (neck not necessarily extended). Head must be above all the neck vertebrae and chest must lift. Adapted from^38^
Neck stretch	Neck is elongated either vertically, horizontally, or diagonally and stretched to its full length. You can typically see the skin underneath the feathers when this occurs. Adapted from^38^
Look	When a bird is swiveling its head to scan the arena. Must turn its head 90° to one side and/or 45° to both sides successively. Look ends when the bird returns their head to neutral position (front) or initiates any other behavior. Adapted from^38^

The behaviors can occur simultaneously, and at least one needs to be observed for the bird to be considered vigilant.

### Statistics

We assessed the inter-observer reliability for latency to feed among the four observers based on video-recorded tests of 12 birds, using Cronbach’s α in JMP Pro 16 (SAS Institute, Cary, NC, Unites States). Intra-observer reliability for the observer scoring vigilance was assessed based on 12 other video-recorded birds, using Cronbach’s α in JMP Pro 16. We fitted generalized linear mixed models (GLMM) to predict the probability of feeding and the latency to step as functions of strain, weight, age, and enrichment environment. Preliminary analyses showed that weight and age were strongly correlated $({R}^{2}=0.74)$. When included in the same model, weight explained more of the variation in bird behavior and thus only weight was included as a predictor in the final list of candidate models. All models included pen and round as nested random effects. We employed logistic regression for data on whether birds fed (yes/no) and linear regression for the latency to step. For both analyses we first fitted models with single predictors of the response variable. We then considered candidate models with pairwise interactions between the single predictor that explained the most variation and all other predictors. We adopted this approach to reduce the likelihood of overfitting. For each analysis, models were compared using AIC and AIC weights^53,54^, and parameter estimates are reported (β).

We fitted an additional GLMM to predict vigilance behavior as a function of bird characteristic (strain, weight, age) and environmental variables (complexity treatment). Owing to a high noise-to-signal ratio, data on the time spent vigilant were first converted to a binary trait (high/low vigilance) and then fitted using a logistic model. We used the mean percentage of time spent vigilant (80% of the test period spent vigilant) as the cutoff for low and high vigilance and used the same suite of fixed and random effects used in the previous models. Model construction, from simple to complex, and model comparison were both performed in the same way as for the other data. All analyses were conducted in R using the *lme4* package^55,56^.

## Results

The inter-observer agreement for latency to feed during the attention bias test was considered strong, with a Cronbach’s α of 0.81 for the four observers. The intra-observer agreement for vigilance behavior was also considered strong, with a Cronbach’s α of 0.98 for the single observer.

The proportion of birds feeding was highest, the proportion of time spent vigilant was lowest, and the latency to step was lowest in the slow-growing strain raised in complex pens (Table 4). Fast-growing broilers raised in simple pens had the longest latency to step with fewest birds feeding, and most time spent vigilant during the ABT, compared to the other strain or complexity treatments (Table 4).

**Table 4:** Data means and ranges of likelihood to feed, latency to first step, and vigilance behavior in fast- and slow-growing broilers housed in simple or complex pens.

Strain	Complexity	Birds observed feeding		Latency to step (sec)		Time spent vigilant (%)	
		n	%	n	Mode (quantiles)	n	Mode (quantiles)
Fast-growing	Simple	144	28	144	37 (16–83)	44	96 (87–99)
	Complex	135	35	135	31 (10–71)	43	80 (60–98)
Slow-growing	Simple	141	54	141	33 (10–75)	47	76 (67–87)
	Complex	143	58	143	21 (7–38)	43	75 (60–94)

### Likelihood of feeding

**Figure 2:**
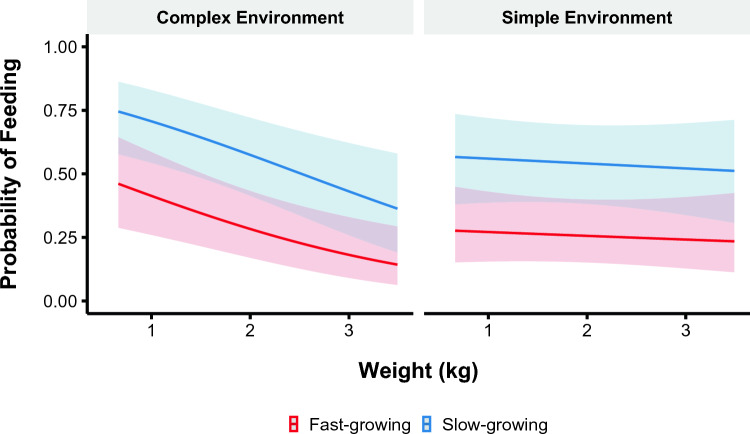
Likelihood of feeding during the attention bias test. The data are displayed for the fast- (red) and slow-growing (blue) strains and complex and simple environments across body weights. The line represents the mean and the shaded areas represent a 95% confidence interval.

We fitted a logistic mixed model to predict the probability of feeding as a function of strain, weight, and environmental complexity. The best supported model included all three predictors and the interaction between weight and environmental complexity (Supplementary Table S1). Slow-growing strains were more likely to feed regardless of weight or environmental complexity (β = 1.23, 95% confidence interval (CI) [0.48, 1.98], p = 0.001). Heavier birds were less likely to feed (β = − 0.58, 95% CI [− 0.94, − 0.22], p = 0.002), and this pattern was more pronounced in complex environments than simple environments (Fig. 2). The interaction between weight and environmental complexity impacted the likelihood to feed (β = 0.50, 95% CI [4.39e−03, 1.00], p = 0.048). The effect of environment on feeding behavior decreased as birds reached the heaviest weights (Fig. 2). At intermediate weights, birds in complex environments were marginally more likely to feed than birds housed in simple environments (β = 1.14, 95% CI [− 0.02, 2.30], p = 0.054).

### Vigilance behavior

**Figure 3:**
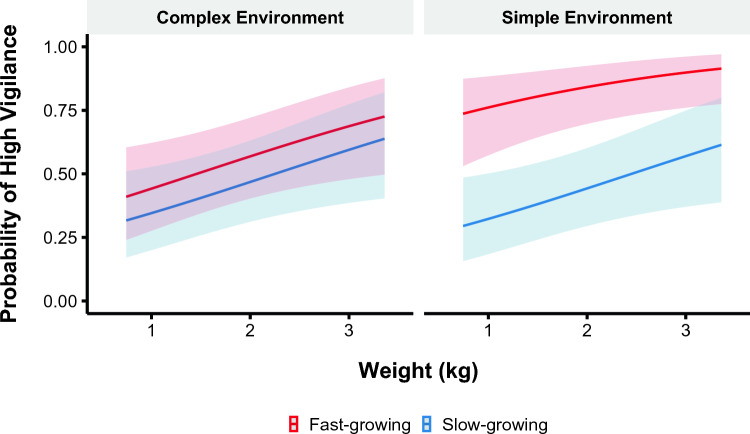
Likelihood of high vigilance behavior (> 80% of test duration) within the attention bias test. The data are displayed for the fast- (red) and slow-growing (blue) strains and complex and simple environments across body weights. The line represents the mean and the shaded areas represent a 95% confidence interval.

We fitted a logistic mixed model to predict the probability of vigilance behavior as a function of strain, weight, and environmental complexity. The top supported model contained strain, weight, and environmental complexity as predictors, as well as the interaction between strain and environmental complexity (Supplementary Table S2; Fig. 3). Broilers were more likely to be highly vigilant as they gained weight regardless of strain or complexity (β = 0.51, 95% CI [0.07, 0.95], p = 0.024). Additionally, a greater proportion of broilers housed in simple environments were highly vigilant compared to in complex environments (β = 1.39, 95% CI [0.34, 2.44], p = 0.009). In simple environments, slow-growing broilers were less likely to be highly vigilant than fast-growing broilers (β = − 1.49, 95% CI [− 2.89, − 0.10], p = 0.036). In contrast, there was no difference between strains housed in complex environments (β = − 0.40, 95% CI [− 1.34, − 0.53], p = 0.39).

### Latency to first step

**Figure 4:**
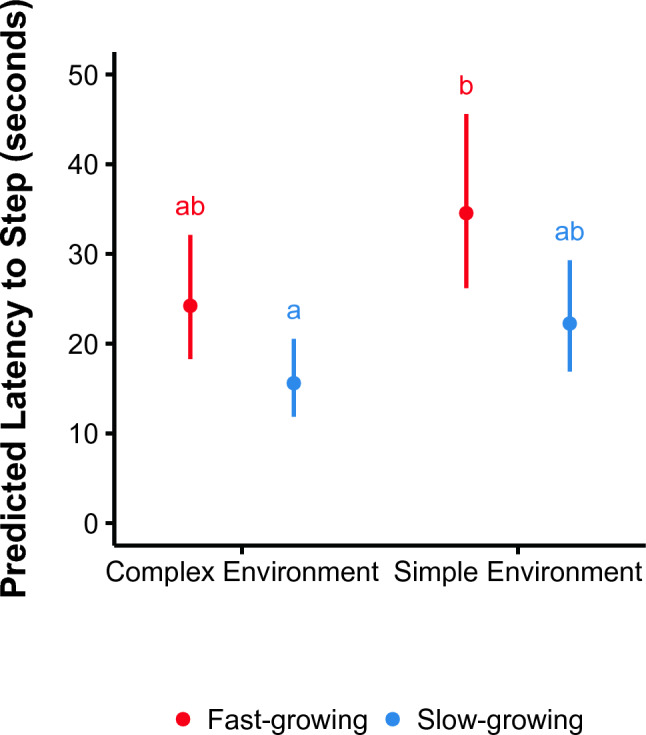
Latency to first step within the attention bias test. The data are displayed for the fast- (red) and slow-growing (blue) strains and complex and simple environments. Whiskers indicate 95% confidence intervals and unique superscripts indicate a difference of p < 0.05.

We fitted a linear mixed model to predict latency to step as a function of strain, weight, and environmental complexity. The best supported model included strain and environmental complexity as predictors (Supplementary Table S3). Slow-growing broilers stepped sooner than fast-growing broilers regardless of environmental complexity (β = − 0.44, 95% CI [− 0.76, − 0.12], p = 0.007). Broilers raised in a complex environment stepped sooner than broilers raised in a simple environment (β = 0.36, 95% CI [0.04, 0.68], p = 0.030). Pairwise comparisons revealed that the only significant difference in step times was between slow-growing broilers raised in complex environments and fast-growing broilers raised in simple environments (Tukey test, p = 0.012; Fig. 4).

## Discussion

Anxiety is an affective state that is beneficial for the survival of an animal. However, if the animal’s anxiety response is inappropriate, then the animal will suffer^57^. We investigated anxiety in fast- and slow-growing broilers as they gained weight and aged, while housed in a simple or complex environment. Both strain and environmental complexity impacted anxiety in broilers, with effects in some cases dependent on body weight. Slow-growing birds were less anxious than fast-growing birds (likelihood to feed; vigilance in simple environments), although they seemed more restless (latency to first step). Light birds were less anxious than heavier birds (likelihood to feed; vigilance) in complex environments and less anxious than light birds in simple environments (likelihood to feed). Complexity reduced anxiety (vigilance).

Fast-growing broilers at 2-kg body weights were tested using a similar test protocol while housed in complex or simple environments^26^. Aligned with our findings, fast-growing broilers were less anxious when housed in complex environments compared to simple environments. Although our results indicate a similar benefit of complexity on anxiety, the proportion of birds feeding did differ between studies, with 64% of birds feeding^26^, compared to 35% of fast-growing birds in the current study at the same weight point. Our response rate of 32% at 1-kg body weight was higher than reported in a pharmacological validation study, where 2% of fast-growing broilers (1.3 kg body weight) treated with an anxiogenic substance began feeding, and 10% of the saline-treated broilers began feeding^49^. The handling and injection procedure may have reduced response rates in their study compared to ours, as rough handling can increase fear in broilers^58,59^.

Other slow-growing broilers that were housed with birds in the current experiment underwent an ABT (at 3 kg body weight) and showed a response rate of 91% in the high-complexity treatment and 80% in the low-complexity treatment^60^. These birds, not used for the data collection reported in the current study, were naïve to the ABT, but did undergo repeated handling and judgement bias training and testing^60^. This may explain the higher response rate in^60^ compared to the 47% of 3-kg slow-growing broilers feeding in this experiment. This suggest that repeated exposure to novel experiences, perhaps especially experiences that stimulate cognition, can contribute to either how birds respond in an ABT, or can reduce bird anxiety.

Slow-growing broilers were overall more likely to begin feeding than fast-growing broilers, indicating they experienced less anxiety. Slow-growing broilers may experience a greater sense of self-efficacy when faced with the threat of a predator. Self-efficacy is defined as an individual’s confidence about their capability to perform a specific task^61^. For broilers in the ABT, this could be translated to their sense of ability to escape a potential predator. Lack of self-efficacy and anxious thoughts and behavior are positively associated in humans^62–67^. This may be similar in other species, including chickens. Due to the genetic selection for meat yield, fast-growing broilers have different body conformation than slow-growing broilers. Breast meat accounts for a greater proportion (35%^68^ 27%^69^) of the carcass yield in fast-growing broilers than slow-growing broilers (25%^68^ 18%^69^). Fast-growing broilers also have a shorter body length (24 cm) compared to slow-growing broilers (30 cm) while having similar breast muscle length (fast-growing 18 cm vs slow-growing 19 cm)^69^. These body composition differences result in more exertion required for locomotion and other physical activities, thus making locomoting require more energy^70^ compared to a chicken with smaller breast muscles and larger body length. We theorize that fast-growing broilers would experience a lack of self-efficacy because they consider their own difficulty escaping an actual predator based on their physical abilities, and therefore experience more anxiety when posed with a threat of a predator, compared to a slower-growing strain with a greater sense of self-efficacy. Since they are less likely to successfully escape, they may feel more anxious when faced by this situation.

Further impacting their sense of self-efficacy could be the birds’ gait. In the ABT, broilers were required to move towards the feed in order to eat. Fast-growing broilers experience worse gait^7,71^ and expend more energy to locomote than slow-growing broilers^70^, potentially impacting their willingness to eat during the test. However, only non-lame broilers were selected for testing and broilers only needed to take a maximum of three steps to access the feed. Thus, differences in gait should not have impacted eating behavior during the test.

Fast-growing broilers were less likely to be highly vigilant when raised in a complex environment compared to fast-growing broilers in a simple environment. Similarly, anxiety was reduced in fast-growing broilers raised with enrichments^26^ and in starlings offered water baths^72^ compared to a barren control. Access to resources can improve health and affect, improving leg strength^19,73^, walking ability^20–23^, and increase optimism^29^ in fast-growing broilers. These physical and psychological benefits of environmental complexity may have contributed to the fast-growing broilers’ sense of self-efficacy, resulting in their reduced vigilance, thus decreased anxiety during the test. This is similar to how increased self-efficacy reduced anxiety in humans^67^. Fast-growing broilers from complex pens may have expected a more positive outcome than fast-growing birds from simple pens, thus leading the former to be less vigilant. When broilers are placed into the ABT arena, they are faced with a novel environment and novel feed, which can induce a fear response. Environmental complexity can reduce fearfulness^25,26,28^. As such, birds from the complex environment may have been less fearful in the novel ABT environment and more willing to interact with a novel food item than birds from the simple environment. However, other studies have not found an effect of complexity on fearfulness^25,26,74,75^. The ABT likely induces both fear and anxiety, and fast-growing broilers from complex pens seemed to be better equipped to cope with these conditions compared to those from simple pens.

Anxiety was mitigated by complexity at lower weights, but increased and became similar in birds from simple and complex environments at later weight points. Lighter broilers in a complex environment were more likely to begin feeding compared to heavier broilers in a complex environment, with no difference in the simple environment. This indicates less anxiety for light compared to heavy broilers when raised in a complex environment only. This suggest that the environmental resources were beneficial in reducing anxiety for all broilers, but only at low body weights. The resources may not have been suitable for heavier birds even if motivation to use the resources remained. The fast-growing broilers may not have been able to benefit from the resources as they gained weight due to their reduced ability to interact with them^76^. Yet, slow-growing broilers do not experience such a reduction in ability^7,71^, while the same effects of weight gain were observed. Rather, the reduced benefit with increasing weight may be related to their age when gaining weight, as they begin to mature and develop social hierarchies. In White Leghorn × Rhode Island Red chickens, males may begin aggressive pecking as early as two weeks of age, but reach adult frequencies of pecking between 8 and 10 weeks of age^77^. The slow-growing broilers were 8 weeks old at 3 kg live weight, thus it is possible that a similar behavioral development took place, although we cannot confirm this based on the data or other research. This may have increased agonistic behavior related to available resources, compared to the fast-growing broilers that were less than 6 weeks old at 3 kg live weight. The increased social pressures may have contributed to anxiety at later ages, especially in complex environments where resources were limitedly available, in contrast with simple environments where no resources were available besides feed, water, and litter. This could imply that slow-growing broilers may need more resources as they grow older in order to avoid potential negative social interactions and subsequent negative affect. This argumentation is not supported by previous work. Slow-growing broilers did not show increased conspecific aggression with age, although the authors mention methodological limitations to determine this conclusively^78^. It would be valuable to determine if complexity induces negative social interactions as birds mature, and if a greater availability of resources would limit this potential impact^4–6,78–85^.

Slow-growing broilers had a shorter latency to step in complex environments compared to fast-growing broilers in simple environments only. Anxiety in chickens is characterized by restless behavior, which is typically quantified by a shorter latency to step and an increased number of steps within an open field test^86,87^. Similarly, a short latency to step was associated with anxiety in fast-growing broilers given an anxiogenic drug compared to a saline control^49^. This would suggest that slow-growing broilers in complex environments are more restless, thus more anxious, which is opposite of what other behavioral indicators suggest. However, others reported no differences between treatments (hens^37^; broilers^26^) or applied an opposite interpretation^38^. This suggests that the measure is difficult to interpret. Perhaps, the direction of movement could provide more insight, with movement towards the feeder possibly reflecting less anxiety, and movement away from the feeder a reflection of restlessness and anxiety. Activity and number of steps are recorded in other species, rather than latencies to step (pigs^88^; sheep^89^). Based on these prior studies, and the conflicting interpretations and results, we have determined that latency to first step may not be the optimal measure for anxiety.

Many birds did not eat during the ABT; therefore, we could not statistically analyze the latency to feed. Novelty of the test environment may have limited their behavioral responses due to fear and anxiety, which could be mitigated by habituation prior to testing. Broilers spent 79% of the time performing vigilance behaviors during the ABT, indicating a high level of anxiety. Habituation may reduce fear and anxiety caused by other stimuli than the conspecific alarm call. Previous experiments allowed birds to habituate to the arena^37,38^, although others did not habituate chickens to the arena and still found differences in latency to feed^26,36^. It may be beneficial to habituate the broilers to the arena and the novel feed, depending on genetic strains used or treatments provided. The large proportion of time spent performing vigilance behaviors in this experiment may indicate the high levels of anxiety experienced, explaining why they did not begin eating within the test.

The test duration of 5 min may not be long enough to detect a difference in latency to feed between treatment groups. A prior study used 10 min for the ABT^37^, while others successfully used a 7-min^49^ or 5-min approach^26,36^. A longer testing period could increase participation from anxious broilers.

Our bird selection procedure for good gait may have biased our results to more positive affect. We selected birds because walking ability may confound the results of the ABT, since birds needed to walk to the feeder. Lameness may reduce their willingness to move to eat, appearing more anxious^90^. Gait disorders cause pain in fast-growing broilers^91–94^ and increase fear^95^. Birds with better gait may actually be less anxious than birds with worse gait, as pain is a negative experience with affective components^96^. Experiencing chronic pain can increase anxiety, as reported in humans^97–99^ and rats^100–102^. Therefore, broilers with impaired walking ability were likely experiencing more negative affect. However, our design excluded these animals in the assessment.

This experiment is the first to assess differences in affective state, specifically anxiety, between fast- and slow-growing strains housed with or without additional resources. The improved affect (reduced anxiety) in the slow-growing strain used here was not consistent as birds gained weight (anxiety increased), suggesting that a move to slow-growing strains may require more adjustments to commercial practices besides just choosing a broiler strain with different genetic potential for growth. However, the initially low anxiety in the slow-growing strain does show promise for an animal welfare improvement compared to the fast-growing strain, even though it is only for the first half of life. Future studies should focus on testing the same and other genetic strains to confirm whether our results are representative of the same and other fast- and slow-growing strains.

## Conclusion

Our objective was to determine how fast-growing and slow-growing broilers’ anxiety changed as they gain weight and age, when housed in complex or simple environments. We hypothesized that fast-growing broilers and broilers raised in a simple environment would be more anxious than slow-growing broilers or broilers in a complex environment. Somewhat in line with expectations, fast-growing broilers were more anxious than slow-growing broilers regardless of environmental complexity or weight. A complex environment reduced anxiety for the first half of life in both strains. We also hypothesized that anxiety would increase in fast-growing broilers, as they aged and gained weight, but not in slow-growing broilers. This was partially true, with both strains becoming more anxious as they gained weight. Producers may improve broiler chicken affective state by using slow-growing strains, processing these broilers at lower weights (before anxiety increases), and by increasing environmental complexity.

### Supplementary Information

Supplementary Information.

## Data Availability

Data underlying this manuscript is made available through the Virginia Tech Data Repository at https://doi.org/10.7294/25196813.
