# Supplementary material for: Environmental complexity impacts anxiety in broiler chickens depending on genetic strain and body weight
Source: Sci Rep. 2024 Jul 30;14:17535. doi: 10.1038/s41598-024-67965-z (PMC11289402; doi:10.1038/s41598-024-67965-z)
Supplement: Supplementary file 1 — Supplementary Information. [file 41598_2024_67965_MOESM1_ESM.pdf]

Supplementary table 1. comparison of logistic regression models describing the probability of high vigilance (>80% time spent vigilant) as a function of bird weight, strain, and enrichment environment. All models included round and pen as nested random effects. Models were compared using AIC and AIC weights (w_i).

Model	AIC	ΔAIC	w_i
$\sim weight + strain \times EE$	230.9	0.0	0.60
$\sim weight + strain$	233.5	2.6	0.16
$\sim strain \times EE$	234.2	3.3	0.11
$\sim weight \times strain$	235.5	4.6	0.06
$\sim strain + EE$	236.5	5.6	0.03
$\sim strain$	237.2	6.3	0.03
$\sim weight$	239.1	8.2	0.01
~ 1	242.6	11.7	0.00
$\sim EE$	242.6	11.7	0.00

Supplementary table 2. comparison of logistic regression models describing the probability of feeding as a function of bird weight, strain, and enrichment environment. All models included round and pen as nested random effects. Models were compared using AIC and AIC weights (w_i).

Model	AIC	ΔAIC	w_i
$\sim strain + weight \times EE$	707.8	0.0	0.34
$\sim strain + weight$	708.2	0.4	0.29
$\sim strain \times weight$	709.1	1.3	0.18
$\sim strain + weight + EE$	709.7	1.9	0.13
$\sim strain$	712.9	5.1	0.03
$\sim strain + EE$	714.5	6.7	0.01
$\sim weight$	715.0	7.2	0.01
$\sim strain \times EE$	716.4	8.6	0.00
~ 1	719.7	11.9	0.00
$\sim EE$	721.3	13.5	0.00

Supplementary table 3. comparison of regression models describing the latency to step as a function of bird weight, strain, and enrichment environment. All models included round and pen as nested random effects. Models were compared using AIC and AIC weights (w_i).

**Model**	$\boldsymbol{AIC}$	$\boldsymbol{\Delta AIC}$	$\boldsymbol{w_i}$
$\sim strain + EE$	1893.0	0.0	0.38
$\sim strain$	1893.7	0.7	0.27
$\sim strain \times EE$	1895.0	2.0	0.13
$\sim EE$	1895.8	2.8	0.09
~ 1	1896.0	3.0	0.08
$\sim strain + EE + weight$	1898.1	5.1	0.03
$\sim EE + weight$	1901.0	8.0	0.01
$\sim weight$	1901.1	8.1	0.01
$\sim EE \times weight$	1903.7	10.7	0.00
